# From Dermatology to Nephrology: Unveiling Granulomatosis With Polyangiitis

**DOI:** 10.7759/cureus.89554

**Published:** 2025-08-07

**Authors:** Shazia Hussain Miranda, Rafael Davila Pacheco, Elizabeth Zaragoza Ramírez, Kelly Andrea Arenas Sánchez, Delfino Eduardo Ordaz Velázquez

**Affiliations:** 1 Internal Medicine, Centro Medico Nacional 20 de Noviembre, Mexico City, MEX; 2 Internal Medicine, Hospital Regional Lic. Adolfo Lopez Mateos, Mexico City, MEX; 3 Dermatology, Centro Dermatológico “Dr. Ladislao de la Pascua", Mexico City, MEX; 4 Internal Medicine, Hospital General Dr. Dario Fernandez Fierro, Mexico City, MEX

**Keywords:** cytomegalovirus (cmv), leucocitoclastic, necrotizing vasculitis, pauci-immune glomerulonephritis (gn), pr3-positive granulomatosis with polyangiitis

## Abstract

Granulomatosis with polyangiitis (GPA), formerly known as Wegener’s granulomatosis, is a rare form of pauci-immune vasculitis that primarily affects the respiratory tract and kidneys, though it can involve virtually any organ system. As a systemic vasculitis, it targets small- and medium-sized blood vessels and is associated with anti-neutrophil cytoplasmic antibodies (ANCA), particularly those directed against proteinase 3 (PR3). Due to its nonspecific symptoms and variable clinical presentation, GPA requires a high index of suspicion for timely diagnosis. Early treatment is essential, as the disease carries a high risk of morbidity and mortality if left untreated. We present a case of a patient with a one-year history of cutaneous lesions who was ultimately diagnosed with GPA, with an unusual concurrent infection by cytomegalovirus (CMV).

## Introduction

The three main anti-neutrophil cytoplasmic antibodies (ANCA)-associated vasculitides, as defined by the 2012 Chapel Hill Consensus Conference on the Nomenclature of Systemic Vasculitides, are granulomatosis with polyangiitis (GPA), microscopic polyangiitis (MPA), and eosinophilic GPA (EGPA). Among these, GPA is the most common, with a global prevalence of 12-14 cases per million people and a peak incidence between the ages of 40 and 65 years [1].

The first case was described in 1931; however, it was properly identified as a vasculitis in 1936 by the pathologist Friedrich Wegener. GPA is a necrotizing vasculitis that affects small- and medium-sized blood vessels and can, therefore, involve virtually any organ. Nevertheless, it most frequently affects the respiratory tract (approximately 90%), kidneys (70%-85%), and skin (30%-50%), with renal involvement being the major prognostic factor. Patients presenting with palpable purpura have a higher likelihood of renal involvement, often demonstrating leukocytoclastic vasculitis on histopathological examination [2]. The pathogenic hallmark of GPA is the loss of immune tolerance by T and B cells to neutrophil-derived proteins - primarily proteinase 3 (PR3) or myeloperoxidase (MPO) - leading to inflammation of vessel walls and the formation of peri- and extravascular granulomas [3].

To date, there are no universally established diagnostic criteria or specific tests for GPA. As such, diagnosis is primarily based on clinical presentation, supported by laboratory findings, imaging studies, ANCA serology, and histopathological confirmation when available.

## Case presentation

A 55-year-old man presented to the emergency department in March 2025 with a two-week history of cutaneous lesions on his arms and legs, associated with a non-productive cough, intermittent fever, and nocturnal diaphoresis. On arrival, his vital signs were as follows: heart rate 81 bpm, blood pressure 103/67 mmHg, respiratory rate 18 breaths/minute, oxygen saturation 80% on room air, and temperature 36.5 °C. Physical examination revealed bilateral basal fine crackles on lung auscultation and painless purpuric skin lesions on the upper and lower extremities, as well as the abdomen. No other abnormalities were noted on examination.

A chest radiograph revealed multiple pulmonary nodules and a ground-glass appearance, predominantly in the right lung field. Initial laboratory investigations showed leukocytosis with neutrophil predominance, microcytic hypochromic anemia, and an elevated procalcitonin level. Empirical treatment with intravenous cephalexin and dexamethasone was initiated for a presumed bacterial infection.

**Figure 1:**
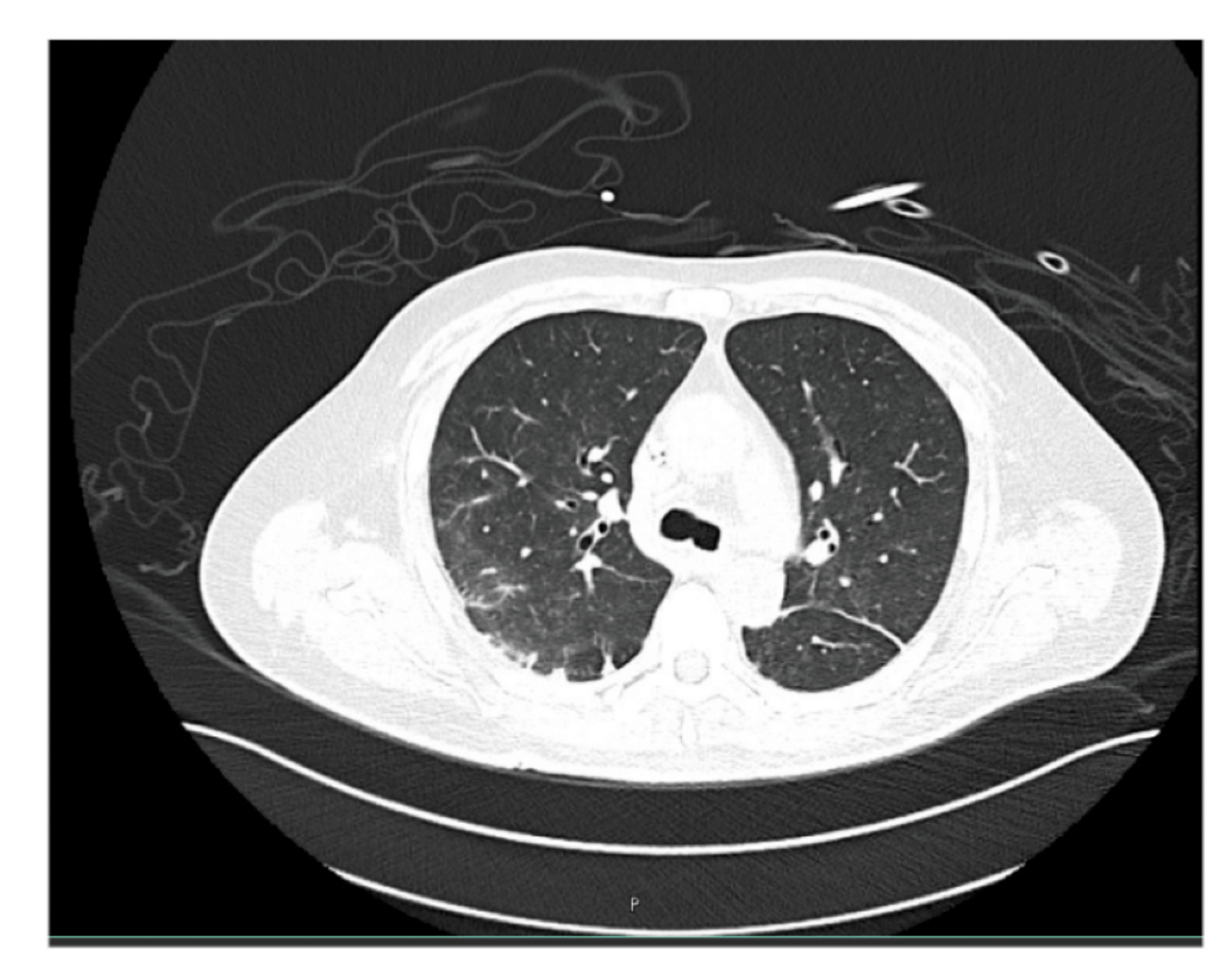
Axial CT scan of the upper lobes. Micronodules in a centrilobular distribution with a tree-in-bud pattern, predominantly in the right lung, along with dilated bronchi in the upper lobes.

**Figure 2:**
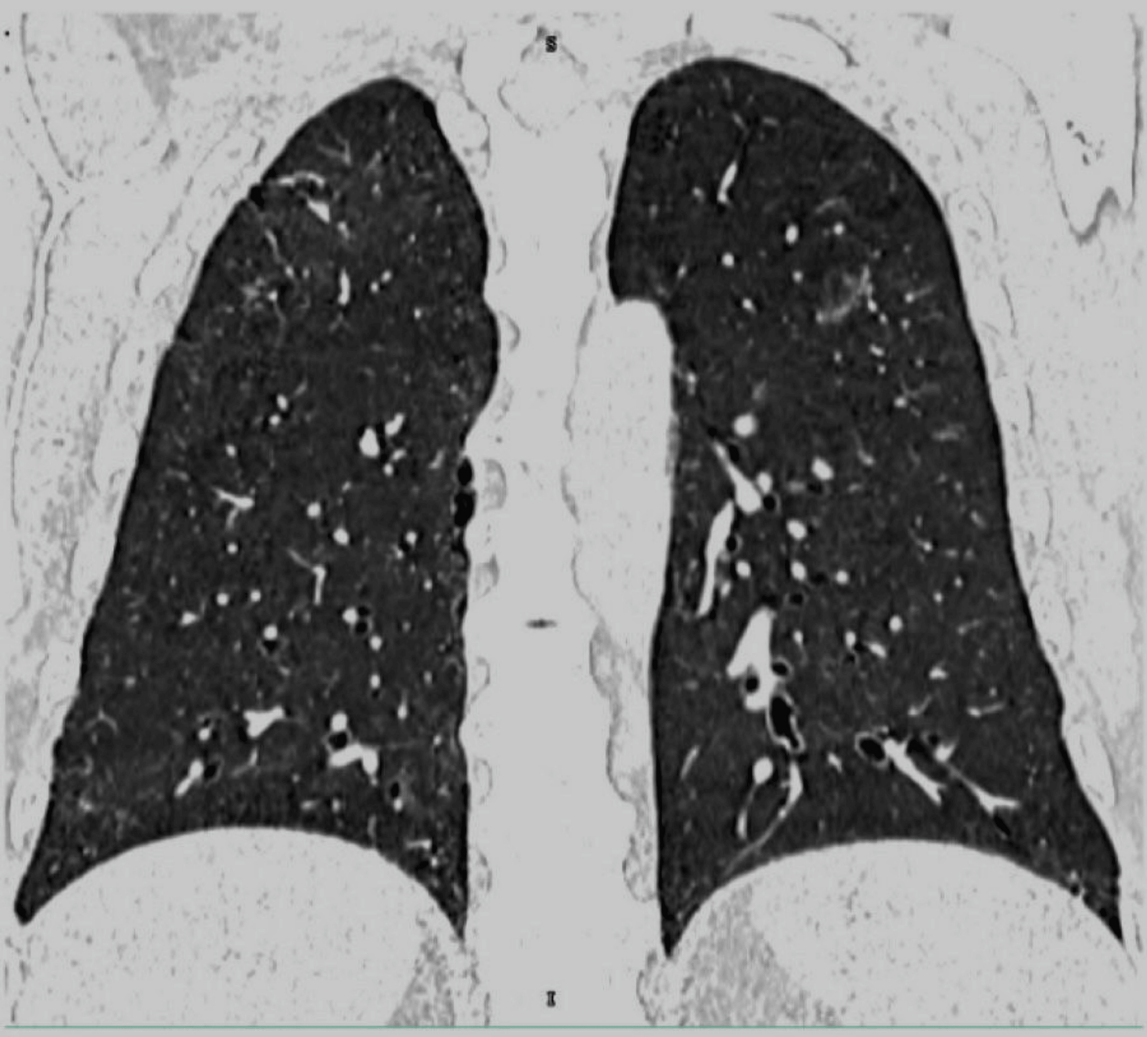
Coronal CT scan of the thorax showing diffuse solid micronodules in both lungs.

**Figure 3:**
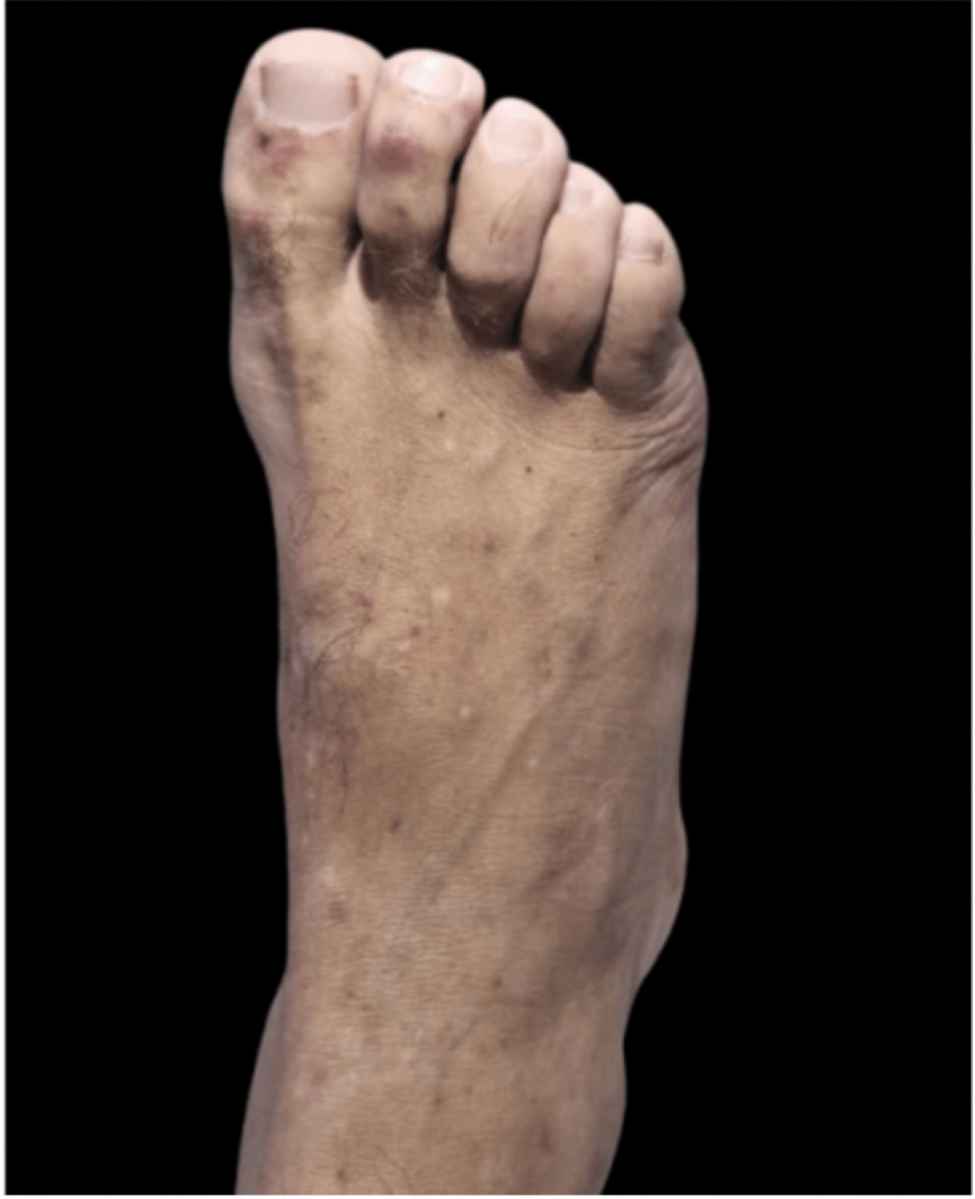
Dorsal left foot: multiple palpable purpura scattered over the dorsal aspect.

Given the constitutional symptoms, including weight loss and persistent fever, Mycobacterium tuberculosis infection was considered. However, all microbiological studies, including acid-fast bacilli staining, culture, and polymerase chain reaction (PCR) testing, returned negative results, effectively excluding tuberculosis as the underlying cause. Subsequently, the patient experienced two episodes of hemoptysis. A CT scan was performed, revealing peripheral areas of ground-glass opacity, a reticulonodular pattern primarily in the right lobe, and multiple bilateral lung nodules (Figures 1-3). Due to the location of the nodules and the patient’s deteriorating condition, a biopsy was deferred.

**Figure 4:**
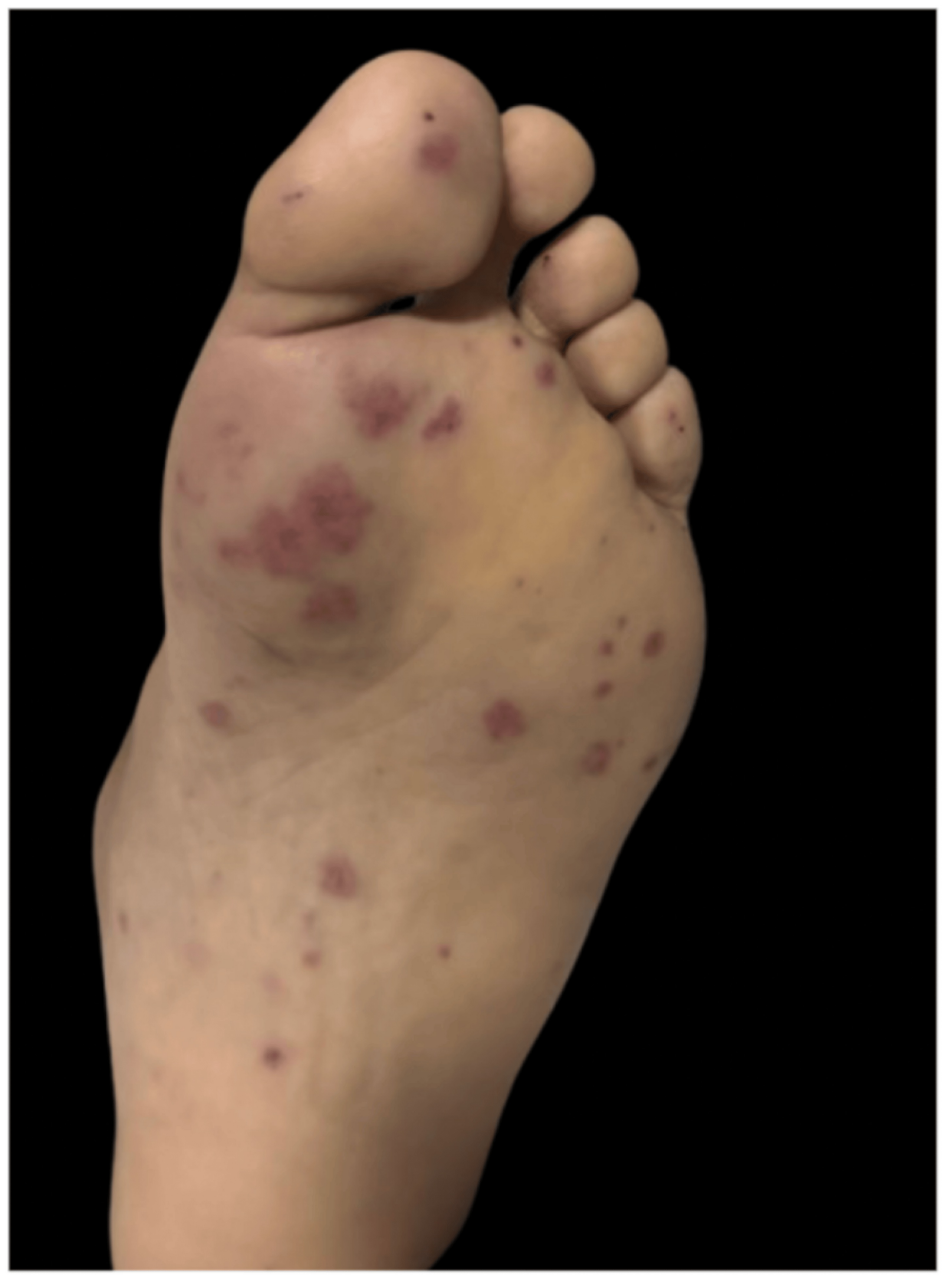
Plantar left foot.

**Figure 5:**
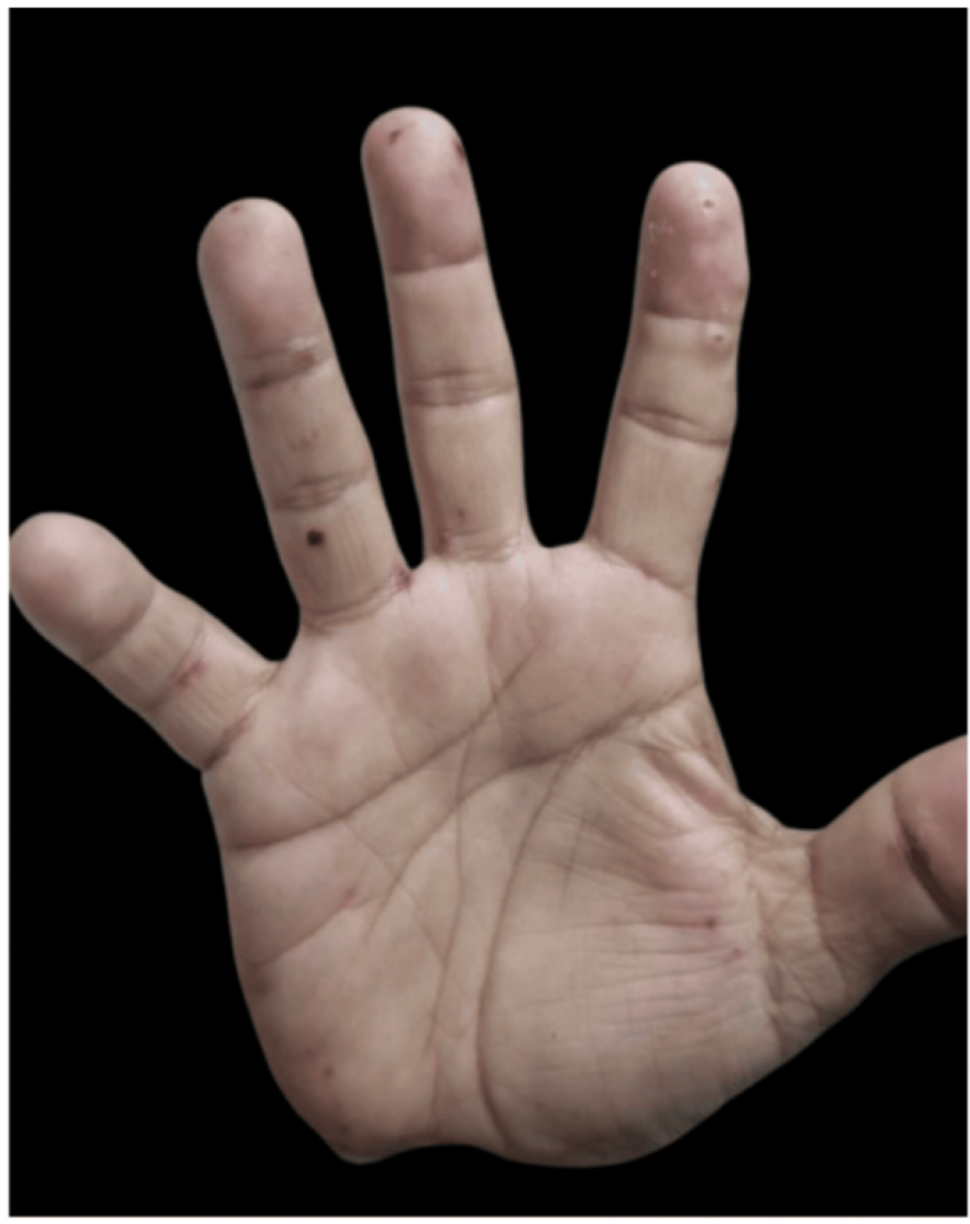
Left palm: palmar surface with palpable purpura distributed mainly on the fingertips and along the palmar creases.

Since admission, the patient developed asymmetrically distributed, painless purpuric cutaneous lesions involving the upper and lower extremities, abdomen, and notably the palms and soles (Figures 3-5). Given these dermatologic findings, a skin biopsy was performed, revealing leukocytoclastic vasculitis involving the small vessels.

Given the vasculitic presentation, a comprehensive workup was initiated to identify potential secondary causes. A TORCH panel and viral serologies, including HIV screening, were obtained. Serologic testing was positive for cytomegalovirus (CMV) IgM antibodies, suggesting a recent or ongoing infection. To further evaluate the degree of viral replication, CMV viral load testing was performed, revealing significantly elevated levels, approximately 10-fold above the normal range, with a quantified value of 1,150 copies/mL. Treatment was promptly initiated and adjusted according to the patient’s renal function. The patient received three consecutive daily doses of intravenous methylprednisolone at 1 g per dose to control the inflammatory process. This was followed by antiviral therapy with valganciclovir 450 mg administered every 48 hours, titrated based on renal clearance (Table 1).

**Table 1:** Renal function test. Renal function tests were conducted at admission, during hospitalization, and after initiation of treatment with cyclophosphamide. BUN, blood urea nitrogen

Date	Glucose (mg/dL)	BUN (mg/dL)	Urea (mg/dL)	Creatinine (mg/dL)	Na (mmol/L)	K (mmol/L)	Cl (mmol/L)
Reference range	70-99	7-20	15-40	0.6-1.3	135-145	3.5-5.0	98-107
At the time of admission	92	107	230.5	9.58	137	7.18	102.2
March 7, 2025	147	103	221.4	10.48	144	5.69	102.2
March 9, 2025	98	121	259.0	11.72	134	5.3	93.3
March 10, 2025	127	148	317.0	12.8	132	5.07	88.0
After initiation of treatment	148	91	195.7	3.48	122	4.6	88.8

A review of the patient’s previous medical records showed a baseline serum creatinine of 2.07 mg/dL and a cystatin C level of 6.27 mg/L, indicating a rapid decline in renal function. Urinalysis revealed macroscopic hematuria, proteinuria, and the presence of dysmorphic red blood cells. These findings raised suspicion for rapidly progressive glomerulonephritis (RPGN); thus, a renal biopsy was done. Histopathological examination of the biopsy demonstrated global glomerulosclerosis, extracapillary proliferative lesions, and crescentic fibrinoid necrosis, consistent with a diagnosis of mixed proliferative glomerulonephritis with interstitial fibrosis and tubular atrophy. Immunofluorescence was positive for fibrinogen but negative for immune complex or complement deposition, supporting a pauci-immune pattern.

Immunological studies revealed decreased complement C3 levels, positive rheumatoid factor, and c-ANCA positivity with elevated anti-proteinase 3 (PR3) antibodies, measured at 763.59 arbitrary antibody units (AAU)/mL, confirming the diagnosis of pauci-immune vasculitis.

Immunosuppressive therapy was initiated with cyclophosphamide, following an induction protocol consisting of a cumulative dose of 2,250 mg administered at weeks 0, 2, 4, 7, 10, and 13. The first dose of 750 mg was administered on April 8, 2025, under the supervision of the Nephrology Department.

## Discussion

GPA is a relatively rare c-ANCA-associated vasculitis defined by the Chapel Hill International Consensus. It is primarily characterized by pulmonary-renal syndrome, affecting the lungs in 95% of cases, the kidneys in 80%, and the upper respiratory tract in 75%-90%. Cutaneous involvement occurs in approximately 50% of cases [4]. Based on system involvement, GPA can be further classified into three categories: classical, involving the three main organs; limited, which primarily involves the lungs; and systemic, where the skin, eyes, and peripheral nervous system may also be affected.

The variety and nonspecificity of symptoms often contribute to diagnostic delays. The 2022 American College of Rheumatology (ACR) and European League Against Rheumatism (EULAR) classification criteria for GPA include the following weighted criteria:

(1) Bloody nasal discharge, nasal crusting, or sinonasal congestion (+3)

(2) Cartilaginous involvement (+2)

(3) Conductive or sensorineural hearing loss (+1)

(4) Cytoplasmic ANCA (c-ANCA) or anti-PR3 ANCA positivity (+5)

(5) Pulmonary nodules, masses, or cavitation on chest imaging (+2)

(6) Granuloma or giant cells on biopsy (+2)

(7) Inflammation or consolidation of the nasal/paranasal sinuses on imaging (+1)

(8) Pauci-immune glomerulonephritis (+1)

(9) Perinuclear ANCA (p-ANCA) or anti-MPO ANCA positivity (−1)

(10) Eosinophil count greater than 1 × 10^9 cells/L (−4)

After excluding mimics of vasculitis, a patient diagnosed with small- or medium-vessel vasculitis could be classified as having GPA if the total score is 5 or more points. In recent studies, these criteria demonstrated a sensitivity of 93% and a specificity of 94% [5].

Due to the significant overlap of clinical manifestations with MPA, diagnosis often relies on the detection of specific immune complexes targeting PR3 or MPO. Antineutrophil cytoplasmic antibody-associated vasculitis (ANCA-associated vasculitis) encompasses a heterogeneous group of autoimmune conditions characterized by necrotizing vasculitis and positive ANCA titers. These titers are reactive to proteinase-3 (PR3-ANCA, also known as C-ANCA) or myeloperoxidase (MPO-ANCA, or p-ANCA). These vasculitides can affect arterioles, capillaries, or venules, involving any organ system with varying degrees of severity.

In our case, the patient presented with palpable purpura as a cutaneous manifestation of GPA, which is a rare initial presentation of the disease. Histopathologically, vasculitis is the typical finding in palpable purpuric lesions, with leukocytoclastic vasculitis being a common pattern. Cutaneous lesions in GPA are diverse, and their development may indicate a relapse of the disease, often accompanied by a concomitant rise in anti-PR3-ANCA levels, as observed in our patient [6].

Pulmonary involvement in GPA ranges from cough and dyspnea to hemoptysis, with alveolar hemorrhage being a major cause of morbidity and mortality. Our patient exhibited features consistent with RPGN, with immunological studies showing negative results except for a positive PR3-ANCA. Renal biopsy revealed crescentic glomerulonephritis with extraglomerular cellular infiltrates, confirmed by immunofluorescence. Electron microscopy was not performed due to unavailability at our institution.

RPGN is a medical emergency requiring rapid diagnosis and prompt treatment, with immunosuppression as the cornerstone of management. Recent guidelines from the ACR and EULAR recommend classifying the disease into either organ- or life-threatening or non-organ/life-threatening categories. Organ- or life-threatening features include glomerulonephritis, pulmonary hemorrhage, cardiac involvement, or retro-orbital disease. The recommended initial treatment involves high-dose corticosteroids combined with induction therapy using cyclophosphamide or rituximab, especially in cases of relapse.

In our case, the patient’s renal function and cutaneous lesions improved dramatically after initiation of treatment. Additionally, trimethoprim-sulfamethoxazole was administered for prophylaxis, following current guidelines. The patient remains under the ongoing care of the Nephrology Department.

## Conclusions

Our patient presented with features characteristic of the systemic form of GPA, with palpable purpura as the initial manifestation, accompanied by rapid deterioration of renal function and nonspecific respiratory symptoms. A skin biopsy was performed before suspicion of vasculitis, guiding us to investigate a clinical entity that is uncommon and often overlaps with various autoimmune conditions. While ANCA serology is essential for diagnosis, it should not be the sole basis; biopsy remains the gold standard for confirming GPA. The diagnostic yield of the kidney biopsy can reach up to 91%, although results should not delay the initiation of treatment. Due to its multisystemic nature, the differential diagnosis is broad, and common causes must be carefully ruled out.

In our patient, considering age, infectious causes, particularly infective endocarditis and tuberculosis, and metabolic and autoimmune conditions were investigated and excluded. However, coinfection can occur, as in this case, and appropriate treatment was accordingly administered. Given the overlap of clinical manifestations, even among pauci-immune vasculitides, serological and histopathological tests, along with established diagnostic criteria, were essential for making an early diagnosis and initiating therapy to improve the patient’s clinical outcome. It is crucial to consider each sign and symptom meticulously, as every detail can contribute to the diagnosis. In this case, the cutaneous manifestations provided valuable clues toward diagnosis.

According to previous reports, palpable purpura is more frequently observed in males and is associated with renal involvement. To date, only one case has been reported where palpable purpura appeared two weeks before renal involvement.

This case underscores the importance of a multisystemic approach and thorough physical examination in diagnosis. Although the association between skin and kidney involvement has not been extensively studied, cutaneous lesions have been reported in 70% to 90% of patients who are seropositive for PR3-ANCA. Therefore, cutaneous manifestations may serve as a valuable indicator for systemic involvement and predictors of renal damage.
